# Maternal Anaemia and Congenital Heart Disease in Offspring: A Case–Control Study Using Linked Electronic Health Records in the United Kingdom

**DOI:** 10.1111/1471-0528.18150

**Published:** 2025-04-23

**Authors:** Manisha Nair, Cynthia W. Drakesmith, Margaret Smith, Clare R. Bankhead, Duncan B. Sparrow

**Affiliations:** ^1^ National Perinatal Epidemiology Unit Nuffield Department of Population Health, University of Oxford Oxford UK; ^2^ Nuffield Department of Primary Care Health Sciences University of Oxford Oxford UK; ^3^ Department of Physiology, Anatomy and Genetics University of Oxford Oxford UK

**Keywords:** anaemia, case–control studies, clinical practice research datalink, congenital heart disease, haemoglobin, risk factor

## Abstract

**Objective:**

Assessment of whether maternal anaemia in early pregnancy is associated with offspring congenital heart disease (CHD).

**Design:**

Matched case–control study.

**Setting:**

January 1998–October 2020, United Kingdom.

**Population:**

Women with a haemoglobin measurement in the first 100 days of pregnancy and a CHD‐diagnosed child.

**Methods:**

Data were extracted from the United Kingdom Clinical Practice Research Datalink GOLD database of electronic health records. Cases were 2,776 women with a CHD‐diagnosed child. These were compared to 13 880 matched controls, women without a CHD‐diagnosed child. Anaemia was classified as < 110 g/L haemoglobin following the WHO definition. A conditional logistic regression analysis was conducted, adjusted for potential maternal demographic and health‐related confounders.

**Main Outcome Measures:**

Offspring CHD diagnosed within 5 years of birth.

**Results:**

123 (4.4%) cases and 390 (2.8%) controls had anaemia. After adjusting for potential confounders, the odds of giving birth to a CHD‐diagnosed child were 47% higher among anaemic mothers (adjusted OR 1.47, 95% CI 1.18,1.83, *p* < 0.001).

**Conclusions:**

The observed association between maternal anaemia in early pregnancy and increased risk of offspring CHD supports our recent evidence in mice. Approximately two‐thirds of anaemia cases globally are due to iron deficiency. A clinical trial of periconceptional iron supplementation might be a minimally invasive and low‐cost intervention for the prevention of some CHD if iron deficiency anaemia is proven to be a cause.

## 1 Introduction

Congenital heart disease (CHD) is a general term for any structural abnormality in the heart present at birth. This is the most common class of birth defects, affecting 1%–2% of live births worldwide [1]. It is a major cause of infant and child mortality and morbidity [2], and often requires ongoing medical assessment and treatment throughout life. CHD results from defects frequently occurring early in the development of the embryo, either because of gene mutation, chromosomal abnormality, or the impact of environmental factors. Despite intensive investigation using next‐generation sequencing technologies, only about 30% of cases can be attributed to a genetic cause [3]. Thus, environmental factors could have a more significant role in causing CHD.

Many environmental risk factors for CHD have been identified using epidemiology or animal studies [4]. Examples are: maternal conditions and diseases, including viral infection and hyperthermia, folate deficiency, pre‐gestational diabetes and/or obesity; exposure to drugs such as anti‐convulsants, retinoic acid and thalidomide; and environmental pollutants. Recently, we identified a previously unknown environmental risk factor causing heart defects in an experimental animal model—maternal iron deficiency anaemia [5]. Globally, 36.5% of pregnant women (~32 million) have anaemia [6]. The effects of maternal anaemia on general foetal development in mid‐to‐late pregnancy are well understood [7], but the existing evidence about the association between maternal anaemia and offspring CHD is mixed and weak. We found eight studies that examined the association between maternal anaemia or iron intake during pregnancy and risk of offspring CHD [8, 9, 10, 11, 12, 13, 14, 15], but none of these examined maternal anaemia in early pregnancy. As the heart forms in the developing embryo between weeks three and eight of gestation, it is important to understand the role of maternal anaemia in early pregnancy. Therefore, our objective was to investigate the association between maternal anaemia in early pregnancy (up to 100 days of gestation) and CHD in offspring in a United Kingdom population.

## 2 Methods

### 2.1 Study Design and Data Sources

We performed a matched case–control study using data from the July 2022 release of the Clinical Practice Research Datalink (CPRD) GOLD database. We were granted access to the datasets through the standard CPRD application process. CPRD is a person‐level database which includes anonymised primary care records for more than 21 million patients from 986 GP practices in the UK [16, 17]. The data are broadly representative of the UK population in terms of sex, age, and ethnicity [17]. All coded primary care information for each patient was available in the dataset, including an up to standard (UTS) date which is assigned to a CPRD practice when the data recorded by the practice are of an acceptable research standard.

For this analysis we used: the Pregnancy Register, which contains a list of pregnancy episodes within the CPRD GOLD database; the Mother‐Baby Link to identify likely mother–baby pairs within the primary care data; linked Hospital Episode Statistics (HES), which contains details of all admissions; cause and date of death from the Office of National Statistics (ONS); and the Index of Multiple Deprivation (IMD). Linked data are only available for practices in England. The methods of linkage and methods of linkage quality evaluation are as described [16, 18].

### 2.2 Participant Inclusion and Exclusion Criteria

We included pregnancies that started between January 1998 and October 2020. No restrictions regarding eligibility for linkage were applied to either mothers or babies. All included cases and controls had ‘research acceptable’ data, defined by a CPRD process that identifies and excludes patients with non‐continuous follow‐up or patients with poor data recording that raises suspicion as to the validity of that patient's record.

We excluded mothers if they did not have any recorded measurement of haemoglobin during the first 100 days of pregnancy, if they had a diagnosis of CHD (to exclude inherited genetic cases), or they had less than one year of UTS data at their current primary care practice at the time of pregnancy.

Information in electronic health records is structured such that each piece of information is assigned a code [19]. CPRD GOLD is coded using Read codes, which are then mapped to CPRD's proprietary medcodes (unique identifiers assigned by CPRD). We collected all the relevant medcodes for each variable into separate code lists (available on our GitHub site [18]).

### 2.3 Selection of Cases and Controls

Cases were mother–baby pairs in which the child had a diagnosis of CHD in the first five years after birth. Controls were mother–baby pairs where the child had not been diagnosed with CHD in the first five years. Mothers' health data were from their primary care records, and presence or absence of CHD in her child was from babies' primary care and HES inpatient and ONS death records. Any evidence of CHD in the baby's health records was determined from a code list based on the International Paediatric and Congenital Cardiac Code [18, 20] and used to identify the cases (summarised in Table S1). The earliest date of any recording of CHD was selected.

Controls were drawn from the remaining pool of pregnancies with no indication of CHD outcome in the offspring. Five controls for each case were identified and matched to cases by the date of start of gestation, as calculated in the Pregnancy Register (± six months). Control babies also had to be contributing data to CPRD in the same year as the incident case was diagnosed. Figure S1 depicts the study population and case–control selection flowchart.

### 2.4 Maternal Anaemia

We extracted all haemoglobin measurements from the mother's health records in the first 100 days of pregnancy. The first recorded haemoglobin value within this time period was included to most closely match our previous animal studies given the timing of human heart development. Valid values were considered between 25 and 185 g/L. Mothers were categorised as anaemic based on a haemoglobin cut‐off < 110 g/L according to the World Health Organisation's definition of anaemia in pregnancy [21], which is used for anaemia management and treatment in the UK.

### 2.5 Confounders

Potential confounders were identified through a review of published literature and expert knowledge. A directed acyclic graph (DAG) was drawn to map all potential confounders (Figure S2), which were factors known to be associated with both the exposure and the outcome. Variables were coded using data from the mother's primary care health records from diagnoses or test results in the first 100 days of pregnancy or before pregnancy start, as appropriate. The potential confounders were:

- Maternal socioeconomic status, measured using linked quintiles of IMD.
- Maternal ethnicity, obtained from linked HES data.
- Maternal age (years) at start of pregnancy (continuous covariate).
- The most recent body mass index (BMI) within one year prior to the start of pregnancy was used to generate BMI categories: underweight (< 20 Kg/m^2^); normal (20–24.9 Kg/m^2^); overweight (25–29.9 Kg/m^2^); and obese (≥ 30 Kg/m^2^). Valid values were considered to be between 10 and 80.
- Maternal smoking (never, ex, current) within a year prior to the pregnancy.
- Heavy maternal alcohol consumption (yes/no) recorded within a year prior to the start of pregnancy.
- Pre‐existing maternal type 1 *diabetes mellitus*.
- Pre‐existing maternal type 2 *diabetes mellitus*.

For variables IMD, ethnicity and BMI, missing data were coded as a separate category. Mothers with missing smoking status were coded as nonsmokers, as patients with missing data are very unlikely to be current smokers [22]; those with missing alcohol status were coded as ‘nonheavy drinkers’, as patients with missing data had similar characteristics to nondrinkers [23]; those with missing information about diabetes were coded as ‘no diabetes’. Where both type 1 and type 2 diabetes classifications were recorded, type 1 diabetes was assumed.

### 2.6 Statistical Analysis

Descriptive statistics such as median and interquartile (IQR) ranges for continuous variables that were not normally distributed and proportions for categorical variables were calculated to compare the characteristics of cases and controls. We conducted conditional logistic regression analyses grouped on matched sets to examine the association between maternal anaemia in the first 100 days of pregnancy and CHD in offspring. We also examined the association between maternal haemoglobin concentration as a continuous variable and CHD in offspring. We also looked at categories of maternal haemoglobin as an exposure. The models were adjusted for all confounding variables listed above. For primary analyses, we used the ‘missing indicator method’, in which observations with missing information are grouped as a separate category, for our primary analysis. Multiple imputation was not done as the proportion of missing data was high and the missing at random assumption may not be valid. Crude and adjusted odds ratios are reported with 95% confidence intervals. A two‐tailed p‐value of < 0.05 was used as the threshold for statistical significance.

We conducted a complete case analysis to compare the findings from the main model. We did additional sensitivity analyses in which we used multiple imputation for BMI. We imputed 40 datasets after first log transforming BMI. All covariates in the analysis model were included in the imputation model, as well as case–control status and pregnancy start date (matching variable). Missing values of IMD and ethnicity were treated as separate categories, as described above. We also repeated multiple imputation and analyses within the group of patients eligible for linkage, as there are very few missing values for IMD or ethnicity in this group.

We calculated the population attributable fraction (PAF) for maternal anaemia in relation to offspring CHD using a re‐expression of Miettinen's formula [24]. Because CHD is a rare condition, we replaced the risk ratios (RR) in the formula with odds ratios (OR).

$${\mathrm{PAF}}_{M}=\left[{\mathrm{\pi OR}}_{u}/1+\pi \left({\text{OR}}_{u}-1\right)\right]\;{\text{OR}}_{c}-1/{\text{OR}}_{c}$$

π is the prevalence of the risk factor in the population; OR_u_ is the unadjusted odds ratio and OR_c_ is the cause or adjusted odds ratio.

All analyses were done using Stata version 18 (StataCorp, College Station, Texas, USA).

### 2.7 Patient Involvement

Neither patients nor the public were involved in the design or analysis of the study. This work uses anonymised data provided by patients and collected by the UK National Health Service as part of their care and support.

## 3 Results

After applying the exclusion criteria, 2,776 mother–baby pairs were included as cases. These were each matched with five controls, giving a total of 13,880 control mother–baby pairs. The most common type of CHD among the included cases was ventricular septal defect (VSD) recorded as a diagnosis in 32% of the cases (*n* = 880), followed by atrial septal defect (ASD) in 23% (*n* = 640) and patent ductus arteriosus (PDA) in 14% (*n* = 376). Information about the types of CHD is presented in Table S1. The descriptive statistics for cases and controls are summarised in Table 1. The median time from pregnancy end to recorded CHD diagnosis was 29 days (IQR 2–120 days). The median maternal age at pregnancy start was 31 years (IQR 26–34 years) for cases and 29 years (25–33 years) for controls. The median timing of maternal haemoglobin measurement from pregnancy start was 71 days (58–84) for cases and 71 days (57–84) for controls. More than 45% of both cases and controls had missing data for BMI. Compared with controls, a higher proportion of mothers grouped as cases were obese despite the median BMI in both groups being 25 kg/m^2^. About 48% of controls had missing data for IMD compared to 31% of cases. Similarly, 49% of controls had missing data for ethnicity compared to 32% of cases. A higher proportion of mothers categorised as cases were also problem drinkers; belonged to Black, Asian and other minority ethnic groups; the most deprived quintile of IMD; or had a diagnosis of type 1 or type 2 diabetes.

**TABLE 1:** Characteristics of the study population.

Characteristics	Statistics are *n* (%) for categorical variables, and median (25 and 75 percentiles) for continuous variables	
	Controls (*n*=13,880)	Cases (*n*=2,776)
Year of pregnancy start	2009.0 (2005.0–2012.0)	2009.0 (2005.0–2012.5)
Age at pregnancy start (years)	29.0 (24.8–33.0)	30.6 (26.2–34.4)
BMI kg/m^2^	25.3 (22.1–29.9)	25.3 (22.0–30.4)
BMI category		
Underweight	709 (5.1%)	164 (5.9%)
Normal	2,706 (19.5%)	569 (20.5%)
Overweight	1,946 (14.0%)	382 (13.8%)
Obese	1,782 (12.8%)	404 (14.6%)
Missing	6,737 (48.5%)	1,257 (45.3%)
Smoking		
Ex‐smoker	1,950 (14.0%)	412 (14.8%)
Never smoker	7,395 (53.3%)	1,528 (55.0%)
Smoker	3,642 (26.2%)	594 (21.4%)
Missing	893 (6.4%)	242 (8.7%)
Problematic drinking		
No	13,447 (96.9%)	2,624 (94.5%)
Yes	433 (3.1%)	152 (5.5%)
Index of Multiple Deprivation		
1 (Least deprived)	1,266 (9.1%)	442 (15.9%)
2	1,347 (9.7%)	375 (13.5%)
3	1,454 (10.5%)	387 (13.9%)
4	1,672 (12.0%)	326 (11.7%)
5 (Most deprived)	1,433 (10.3%)	387 (13.9%)
Missing	6,708 (48.3%)	859 (30.9%)
Ethnicity		
White	6,427 (46.3%)	1,608 (57.9%)
Black	53 (0.4%)	52 (1.9%)
Asian	382 (2.8%)	139 (5.0%)
Mixed	61 (0.4%)	20 (0.7%)
Other	190 (1.4%)	77 (2.8%)
Missing	6,767 (48.8%)	880 (31.7%)
Diabetes^a^		
No diabetes	13,660 (98.4%)	2,665 (96.0%)
Type 1 diabetes	67 (0.5%)	61 (2.2%)
Type 2 diabetes	153 (1.1%)	50 (1.8%)
Time of Hb measurement from pregnancy start (days)	71.0 (57.0–84.0)	71.0 (58.0–84.0)
Haemoglobin g/L	128.0 (122.0–134.0)	128.0 (122.0–134.0)
Hb > = 110 g/L	13,490 (97.2%)	2,653 (95.6%)
Hb < 110 g/L	390 (2.8%)	123 (4.4%)

^a^ Type 2 diabetes excludes people who have codes for both type 1 diabetes and type 2 diabetes, so the categories are mutually exclusive.

The distribution of maternal haemoglobin measurements for cases and controls is shown in Figure S3. The median maternal haemoglobin level in cases was 128 g/L (IQR 122–134 g/L), which was the same as the controls, 128 g/L (IQR 122–134 g/L). However, a higher proportion of mothers in the cases had anaemia defined as Hb < 110 g/dL (123/2776, 4.4%) compared with the control group (390/13880, 2.8%).

The unadjusted analysis showed that the odds of CHD in offspring were 60% higher (OR 1.60; 95% CI 1.30, 97%; *p* < 0.0001) if the mother had anaemia in the first 100 days of pregnancy. The OR was slightly attenuated after adjusting for the confounders, but the odds of offspring CHD were still 47% higher among mothers who had anaemia (adjusted OR 1.47; 95% CI 1.18, 1.83; *p* = 0.0006 (Table 2). We did not find any significant association between maternal haemoglobin concentration as a continuous variable and offspring CHD (Table 2). Results from the analysis of categorical haemoglobin were consistent with an inverse association between the mother's Hb and offspring CHD below the anaemia threshold, but weak or no association above the anaemia threshold (Table S2). The adjusted OR in the complete case analysis was only marginally different from that of the main model (Table S3), but was not statistically significant, as only 14% of the total observations were retained in the regression model for the complete case analysis. Results of other sensitivity analyses around missing data were also consistent (Table S4).

**TABLE 2:** Associations between offspring CHD and maternal anaemia and haemoglobin levels in a UK population.

	Congenital heart defects in offspring			
	Crude OR (95% CI)	*p*	Adjusted OR (95% CI)*	*p*
Anaemia				
No (Hb ≥ 110 g/L)^a^	1		1	
Yes (Hb < 110 g/L)^b^	1.60 (1.30–1.97)	0.0000	1.47 (1.18–1.83)	0.0006
Haemoglobin level (g/L) per 10 g/L	1.00 (1.00–1.00)	0.3094	1.00 (1.00–1.00)	0.3281

^a^ 2,653 cases and 13,490 controls.

^b^ 123 (4.4%) cases and 390 (2.8%) controls.

* Adjusted for potential confounding variables: maternal socioeconomic status, maternal ethnicity, maternal age, maternal BMI, maternal smoking, heavy maternal alcohol consumption, pre‐existing maternal type I and/or type 2 *diabetes mellitus*.

The prevalence of maternal anaemia in the first trimester in our control population was 2.8%, thus we estimate from the PAF calculation that 1.41% (95% CI 0.55, 2.44%) of all CHD cases in this study population could be attributable to maternal anaemia in first 100 days of pregnancy.

## 4 Discussion

### 4.1 Main Findings

This is the first study in a UK population that demonstrates an association between maternal anaemia in early pregnancy and CHD in offspring, demonstrating a 47% higher odds of CHD in the child.

### 4.2 Strengths and Limitations

The primary strength of this study is the large study population from a clinical database and linkage to health records and sociodemographic factors, which allowed us to adjust for many potential confounders. Our study provides more robust evidence compared with other published studies that either did not have haemoglobin measurements or did not assess maternal anaemia in early pregnancy.

The UK National Institute for Health and Care Excellence (NICE) antenatal care guidelines suggest offering a blood test at the first face‐to‐face antenatal appointment by 10 weeks of pregnancy [25]. However, only one‐third of research‐acceptable pregnancies in CPRD had a haemoglobin value recorded in the first 100 days of pregnancy. This may be due to poor record transfer between the electronic records system used by midwives and that used by the mother's GP practice.

While our study establishes proof of concept of the findings of our experimental animal research showing maternal iron deficiency anaemia as a significant factor for offspring CHD^5^, due to limited data, we were unable to assess whether iron deficiency is the cause of anaemia in the study population. Iron deficiency is generally measured using serum ferritin, but this was only available for 8.5% of the cases and 7.9% of controls. There was even more limited availability of other markers of iron, such as transferrin saturation (TSAT) which is an indicator of circulating iron levels in the mother that is available for the foetus through feto‐maternal circulation. Our recent study examining the relationship between anaemia and iron status in pregnant and postpartum women in India using haemoglobin and five different iron biomarkers showed that there is a poor relationship between haemoglobin and the iron biomarkers [26]. In that cohort, around 41% of the pregnant women with anaemia had iron deficiency (ferritin < 15 μg/L), but 17% of nonanaemic women also had iron deficiency, and around 4% of pregnant women with anaemia had high ferritin (> 200 μg/L) [26]. By contrast, a Mendelian Randomisation analysis using UK Biobank data found that genetically predicted higher iron status was associated with a higher level of haemoglobin [27]. Thus, haemoglobin may be a good indicator of iron deficiency in the UK population, but this may not be the case for other population groups in whom anaemia may be due to multiple causes. Furthermore, low haemoglobin could be due to nutritional deficiencies such as Vitamin B12 and folate, as well as a marker of other nutritional deficiencies. However, we did not have data to investigate the association between offspring CHD and other markers of maternal nutritional deficiencies. Therefore, further research is needed to examine whether maternal iron deficiency anaemia or anaemia due to any other cause is associated with an increased risk of CHD in offspring. If maternal anaemia affecting offspring CHD is not found to be just related to iron deficiency, the role of pre‐ and periconceptional supplementation of multiple micronutrients could be considered. This is crucial to plan interventions for reducing maternal anaemia in the periconceptional period (preconception and during the first trimester) which could prevent some types of CHD in the offspring.

Another limitation is that our case–control design cannot establish a causal relationship between maternal anaemia and CHD, although we were able to account for several known confounders. Use of health records reduces misclassification, reporting, and recall bias, but we cannot rule out residual confounding and bias due to large proportions of missing information for some confounding variables. The missing data could bias the association between maternal anaemia and offspring CHD in either direction. However, a sensitivity analysis conducted by rerunning the regression models using imputed data for BMI to control for confounding did not materially change the association between our exposure of interest ‘maternal anaemia’ and the outcome ‘offspring CHD’. The results of the complete case analysis and a sensitivity analysis restricting the models to a population eligible for linkage, and therefore having very few missing values for IMD and ethnicity, are also similar to that of the main model in terms of magnitude and direction of the effect estimate, although the 95% CIs are wider due to a smaller number of observations included in these analyses.

Again, only pregnancies resulting in live births that could be linked to offspring primary care records were included, and not all mothers had a record of haemoglobin in the first 100 days of pregnancy; thus, the possibility of selection bias cannot be ruled out. However, we do not anticipate any of these biases to systematically affect either the cases or the controls.

Embryonic cardiac development occurs between three and eight weeks of gestation, so the ideal measure of exposure would be haemoglobin levels at conception and up to eight weeks (or 56 days) of gestation. Although the median time of haemoglobin measurement in our study population was 71 days of pregnancy (IQR 57–84 days), these measurements are likely to be indicative of haemoglobin at the critical period of gestation. A reverse causality is highly unlikely as CHD in offspring will not influence maternal haemoglobin levels.

Another potential limitation is the lack of information on iron supplementation before or at conception, which we could not account for in our analysis. For example, women planning to conceive might start taking iron and vitamins which were not recorded in the primary care data. Finally, there are at least 30 different subtypes of CHD, and these can arise from distinct morphological and/or molecular mechanisms. Our study did not have sufficient power to determine if any particular CHD subtype(s) were more prevalent in the offspring of anaemic mothers, which could have potentially led to a stronger association between maternal anaemia and these specific subtypes. Therefore, further research is needed. Further research is also required to examine the likely socioeconomic and ethnic disparities in CHD observed in our study as shown in Table 1.

### 4.3 Interpretation (In Light of Other Evidence)

Our findings are comparable to three studies investigating maternal anaemia as a chronic condition and offspring CHD [8, 10, 11]. These studies from Israel, Canada and Taiwan used a clinical coding of anaemia from maternal health records and demonstrated that maternal anaemia was associated with 24% [11], 26% [10] and 31% [8] higher odds of CHD in the child after accounting for other known risk factors. By contrast, two studies suggested that increased haemoglobin [9] or iron levels [13] might increase offspring CHD prevalence. A prospective cohort study from Japan of 91 664 mother–infant pairs with a CHD incidence of 1.38% showed a 10% higher odds of CHD in the offspring per one g/dL increase in maternal haemoglobin in the second trimester of pregnancy [9]. The study did not provide any information about maternal haemoglobin in early pregnancy, so it is difficult to assess whether iron supplementation to anaemic women in early pregnancy caused an increased haemoglobin concentration in the second trimester of pregnancy, leading to a potential reverse causality bias. A case–control study from China that measured iron levels in hair samples of pregnant women found that high maternal iron concentration was associated with nearly three‐fold higher odds of CHD in the offspring [13]. Finally, three studies of iron intake and/or supplementation report mixed effects of low iron levels on offspring CHD prevalence [10, 14, 15]. Another case–control study from the USA did not find any association between CHD in offspring and maternal dietary iron intakes during the periconceptional period assessed using a food frequency questionnaire [9]. Two matched case–control studies from China [14, 15] estimated dietary iron intake and/or iron supplementation during pregnancy by postpartum survey. In both cases they showed a significantly reduced risk of offspring CHD when mothers had higher iron intake throughout pregnancy or took iron supplements perinconceptionally [14, 15]. However, these studies did not assess haemoglobin or iron biomarkers during the critical period of embryonic heart development and may have been subject to recall bias.

## 5 Conclusion

The preliminary evidence generated by our research about the association of maternal anaemia in early pregnancy with increased rates of CHD in the baby has the potential to substantially reduce the number of children born with CHD, currently estimated as ~2.5 million annually [1].

We estimated a PAF of 1.41% (95% CI 0.55, 2.44%) from the 2.8% prevalence of maternal anaemia in our population. However, previous studies of larger UK populations suggest that a better estimate of the overall prevalence of anaemia in the first trimester might be 7%, but could be as high as 13% in some areas [28, 29]. In that case, maternal anaemia might account for between 3.4% and 6.2% of UK CHD cases. Furthermore, the prevalence of maternal anaemia in low‐ and middle‐income countries (LMIC) has been reported to be in excess of 50%. Although it is difficult to extrapolate our results to different populations, similar PAF calculations might suggest that as much as 20% of CHD in LMIC might be attributable to maternal anaemia. LMIC have approximately double the birth prevalence of CHD of the UK [1], and it is possible that maternal anaemia may be an important factor in this difference.

It is now well established that maternal folic acid deficiency early in pregnancy is associated with a four‐fold elevated risk of having a child with a neural tube defect (NTD). A seminal observational study in the 1970s [30], followed by clinical trials in the 1980s [31] demonstrated that periconceptional folic acid supplementation significantly reduces offspring NTD prevalence. Since then, public health recommendations of periconceptional folic acid supplementation and/or mandated folic acid fortification of basic foods have reduced the population prevalence of NTD by 50%–70% [32]. A similar approach may be feasible for maternal anaemia and offspring CHD. It is estimated that two‐thirds of cases of anaemia globally are due to iron deficiency [33]. In many cases, this can be alleviated by iron supplementation [34]. Thus, a clinical trial of iron supplementation before conception and during the first trimester (periconceptional period) could be a minimally invasive and low‐cost intervention for the prevention of some cases of CHD. However, prior to such a trial, validation of our results in a larger observational dataset would be needed to provide evidence of a robust epidemiological association between maternal iron deficiency and offspring CHD.

## Author Contributions

**Manisha Nair:** conceptualisation (supporting); formal analysis (supporting); funding acquisition (supporting); investigation (supporting); writing – original draft preparation (equal); writing review and editing (supporting). **Cynthia W. Drakesmith:** conceptualisation (supporting); data curation (lead); formal analysis (supporting); investigation (equal); software (equal); writing review and editing (supporting). **Margaret Smith:** conceptualisation (supporting); formal analysis (lead); investigation (equal); software (equal); writing review and editing (supporting); visualisation (equal). **Clare R. Bankhead:** conceptualisation (equal); formal analysis (supporting); funding acquisition (supporting); investigation (supporting); project administration (equal); supervision (equal); writing review and editing (supporting). **Duncan B. Sparrow:** conceptualisation (equal); data curation (supporting); funding acquisition (lead); project administration (equal); supervision (equal); visualisation (equal); writing – original draft preparation (equal); writing review and editing (lead).

## Ethics Statement

The Clinical Practice Research Datalink (CPRD) Database was approved by East Midlands–Derby Research Ethics Committee, reference ID 21/EM/0265 (10 Jan 2022). The study obtained ethics approval through the CPRD Research Data Governance process (Protocol Number 22_00183; 20/10/2022).

## Conflicts of Interest

The authors declare no conflicts of interest.

## Supporting information


**Table S1.** Types of congenital heart disease diagnosed in children in the study population.
**Table S2:** Odds ratios (OR) for offspring CHD by maternal haemoglobin category (13,880 controls and 2,776 cases). Adjusted for all covariates.
**Table S3:** Associations between offspring CHD and maternal anaemia and haemoglobin levels in a UK population based on analysis of 821 cases and 1,446 controls with complete covariate data for IMD, ethnicity and BMI.
**Table S4:** Sensitivity analyses for treatment of missing data. Odds ratios (OR) are for CHD in offspring of mothers who had low haemoglobin (< 110 g/L), compared to those with normal haemoglobin levels (≥ 110 g/L). OR were calculated by conditional logistic regression and adjusted for other potential confounders.
**Figure S1**. Study population and case–control selection flowchart.
**Figure S2**. Directed acyclic graph showing the association between maternal anaemia and congenital heart disease in offspring.
**Figure S3**. Population distribution of haemoglobin measurements.

## Data Availability

Data may be obtained from a third party and are not publicly available. This study is based in part on data from CPRD obtained under licence from the UK Medicines and Healthcare products Regulatory Agency (MHRA). The data are provided by patients and collected by the National Health Service (NHS) as part of their care and support. HES Data/ONS Data Copyright 2022, re‐used with the permission of The Health and Social Care Information Centre. All rights reserved. The interpretation and conclusions contained in this study are those of the authors alone. The data that support the findings of this study are available from CPRD, but restrictions apply to the availability of these data, which were used under licence for the current study, and so are not publicly available. Requests to access CPRD data are reviewed via the CPRD Research Data Governance (RDG) Process to ensure that the proposed research is of benefit to patients and public health. More information is available on the CPRD website: https://www.cprd.com/safeguarding‐patient‐data.
