# Supplementary material for: Maternal Anaemia and Congenital Heart Disease in Offspring: A Case–Control Study Using Linked Electronic Health Records in the United Kingdom
Source: BJOG. 2025 Apr 23;132(8):1139–46. doi: 10.1111/1471-0528.18150 (PMC12137751; doi:10.1111/1471-0528.18150)

Supplementary material

Nair *et al*. Maternal anaemia and congenital heart disease in offspring: a case-control study using linked electronic health records in the United Kingdom.

Table S1: Types of congenital heart disease diagnosed in children in the study population

Type	Frequency	%
Ventricular septal defect	880	31.7
Atrial septal defect	640	23.1
Persistent *ductus arteriosus*	376	13.5
Great vessel abnormalities (including TGA)	366	13.2
Unspecified CHD	144	5.2
Outflow tract valve defect	99	3.6
Tetralogy of Fallot	86	3.1
Atrioventricular valve defect	72	2.6
Dextrocardia	22	0.8
Hypoplastic left heart syndrome	15	0.5
Abnormal pulmonary venous return	14	0.5
Atrioventricular septal defect	12	0.4
Unspecified septal defect	11	0.4
Double outlet right ventricle	8	0.3
Unspecified valve defect	8	0.3

Others	23	0.8

* includes all categories with a frequency less than 5: abnormal coronary vessels; double inlet left ventricle; unspecified venous abnormality; congenital heart block; hypoplastic right heart syndrome; *situs inversus*; left ventricular hypertrophy; discordant atrioventricular connection; and persistent *truncus arteriosus*

Table S2: Odds ratios (OR) for offspring CHD by maternal haemoglobin category (13,880 controls and 2,776 cases). Adjusted for all covariates.

			Unadjusted analysis		Multivariate adjusted analysis*	
Hb (g/L)	Controls (n)	Cases (n)	OR (95% CI)	P-value	OR (95% CI)	P-value
0-109	390	123	1.62 (1.31 – 2.01)	0.0000	1.50 1.20 – 1.87)	0.0004
110-119	1,880	355	0.98 (0.86 – 1.11)	0.7045	0.91 (0.79 – 1.04)	0.1695
120-129	5,569	1,080	1.00		1.00	
130-139	4,759	968	1.05 (0.95 – 1.15)	0.3288	1.09 (0.99 – 1.20)	0.0961
≥140	1,282	250	1.01 (0.86 – 1.17)	0.9423	1.09 (0.93 – 1.27)	0.2771

* Adjusted for potential confounding variables: maternal socioeconomic status, maternal ethnicity, maternal age, maternal BMI, maternal smoking, heavy maternal alcohol consumption, pre-existing maternal type I and/or type 2 diabetes mellitus.

Table S3: Associations between offspring CHD and maternal anaemia and haemoglobin levels in a UK population based on analysis of 821 cases and 1,446 controls with complete covariate data for IMD, ethnicity and BMI.

Maternal risk factors	Congenital heart defects in offspring			
	Unadjusted OR (95% CI)	P-value	Adjusted OR (95% CI)*	P-value
Anaemia				
No (Hb ≥110 g/L)†	1		1	
Yes (Hb <110 g/L)††	1.62 (1.02 – 2.56)	0.039	1.38 (0.85 – 2.23)	0.191

† 783 cases and 1,404 controls

†† 38 (4.6%) cases and 42 (2.9%) controls

* Adjusted for potential confounding variables: maternal socioeconomic status, maternal ethnicity, maternal age, maternal BMI, maternal smoking, heavy maternal alcohol consumption, pre-existing maternal type I and/or type 2 diabetes mellitus.

Table S4: Sensitivity analyses for treatment of missing data. Odds ratios (OR) are for CHD in offspring of mothers who had low haemoglobin (<110 g/L), compared to those with normal haemoglobin levels (≥110 g/L). OR were calculated by conditional logistic regression and adjusted for other potential confounders.

Description of sensitivity analysis	Controls (n)	Cases (n)	OR (95% CI)*	P-value
Multiple imputation of BMI. Missing IMD and ethnicity included in imputation model and analysis as separate category	13,880	2,776	1.48 (1.19 – 1.84)	0.0005
Cohort restricted to people with linkage eligibility. Missing BMI (n=3,424), IMD (n=3), ethnicity (n=73) all treated as separate categories in analysis.	5,202	1,836	1.40 (1.03 - 1.89)	0.0317
Cohort restricted to people with linkage eligibility. Multiple imputation of missing BMI (n=3,424). Missing IMD (n=3) and ethnicity (n=73) included in imputation model and analysis as separate categories.	5,202	1,836	1.41 (1.04 – 1.91)	0.0254

* Adjusted for potential confounding variables: maternal socioeconomic status, maternal ethnicity, maternal age, maternal BMI, maternal smoking, heavy maternal alcohol consumption, pre-existing maternal type I and/or type 2 diabetes mellitus.

Supplementary figure list

Figure S1. Study population and case-control selection flowchart.

Figure S2. Directed acyclic graph showing the association between maternal anaemia and congenital heart disease in offspring.

Figure S3. Population distribution of haemoglobin measurements.

Figure S1: Study population and case-control selection flowchart

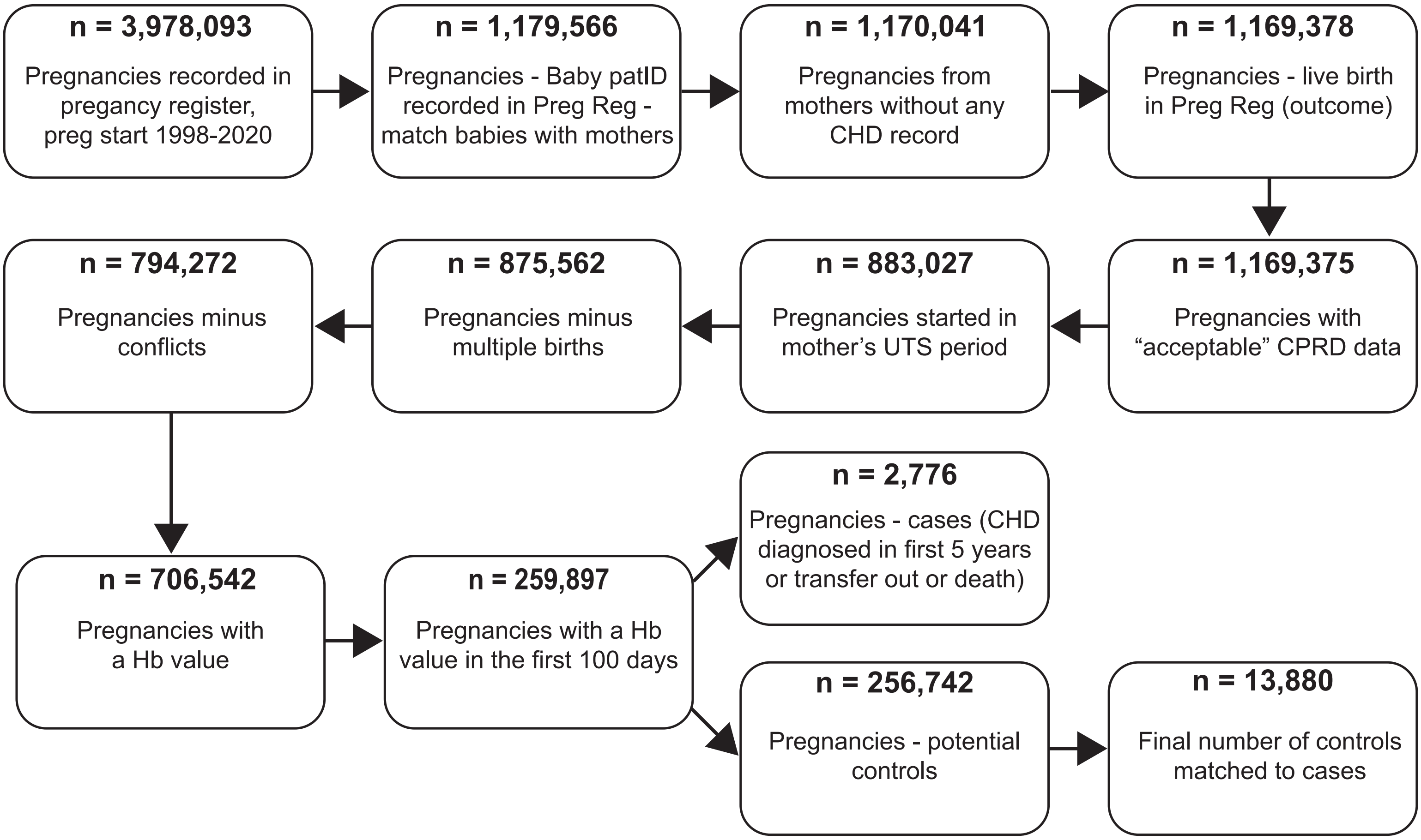

Figure S2: Directed acyclic graph showing the association between maternal anaemia and congenital heart disease in offspring

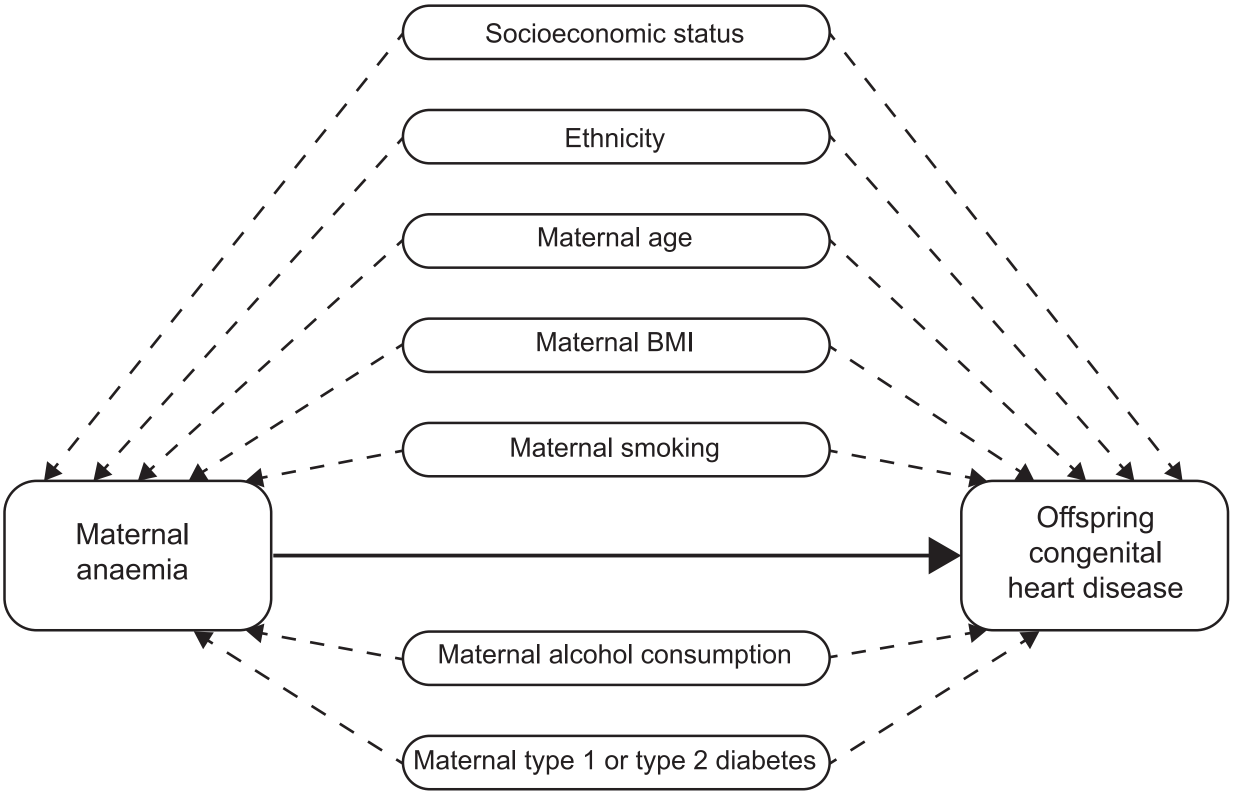

Figure S3. Distribution of haemoglobin measurements

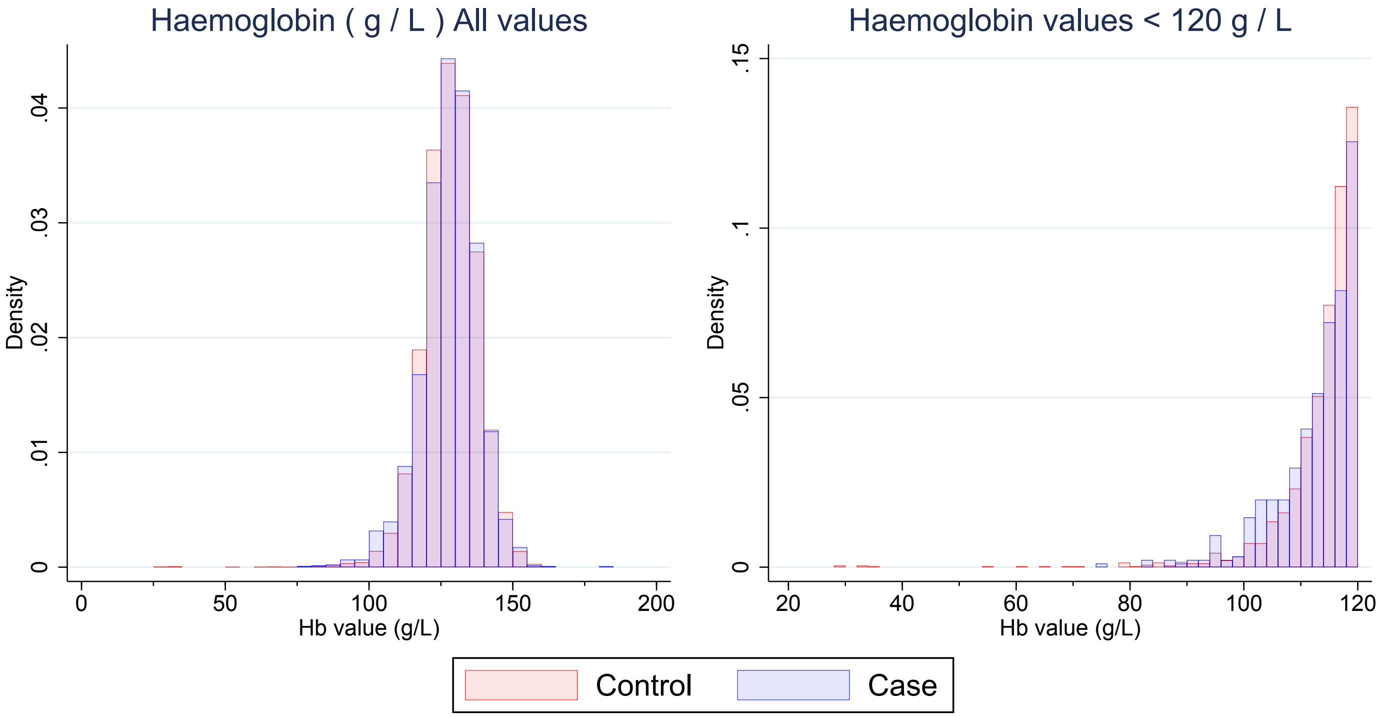

Supplement: Supplementary file 1 — Table S1. Types of congenital heart disease diagnosed in children in the study population. Table S2: Odds ratios (OR) for offspring CHD by maternal haemoglobin category (13,880 controls and 2,776 cases). Adjusted for all covariates. Table S3: Associations between offspring CHD and maternal anaemia and haemoglobin levels in a UK population based on analysis of 821 cases and 1,446 controls with complete covariate data for IMD, ethnicity and BMI. Table S4: Sensitivity analyses for treatment of missing data. Odds ratios (OR) are for CHD in offspring of mothers who had low haemoglobin (< 110 g/L), compared to those with normal haemoglobin levels (≥ 110 g/L). OR were calculated by conditional logistic regression and adjusted for other potential confounders. Figure S1. Study population and case–control selection flowchart. Figure S2. Directed acyclic graph showing the association between maternal anaemia and congenital heart disease in offspring. Figure S3. Population distribution of haemoglobin measurements. [file BJO-132-1139-s001.pdf]
